# Is there a molecular basis for solvent drag in the renal proximal tubule?

**DOI:** 10.1007/s00424-022-02773-w

**Published:** 2022-11-23

**Authors:** Dorothee Günzel

**Affiliations:** Clinical Physiology/Nutritional Medicine, Department of Gastroenterology, Rheumatology and Infectious Diseases, Charité–Universitätsmedizin Berlin, Hindenburgdamm 30, 12203 Berlin, Germany

**Keywords:** Transepithelial transport, Tight junction, Paracellular channel, Claudin protein family

## Abstract

The concept of solvent drag, i.e., water and solutes sharing the same pore and their transport being frictionally coupled, was first proposed in the early 1950s. During the following decades, it was applied to transport processes across cell membranes as well as transport along the paracellular pathway. Water-driven solute transport was proposed as the major mechanism for electrolyte and nutrient absorption in the small intestine and for Cl^−^ and HCO_3_^−^ reabsorption in the renal proximal tubule. With the discovery of aquaporins as transcellular route for water transport and the claudin protein family as the major determinant of paracellular transport properties, new mechanistic insights in transepithelial water and solute transport are emerging and call for a reassessment of the solvent drag concept. Current knowledge does not provide a molecular basis for relevant solvent drag-driven, paracellular nutrient, and inorganic anion (re-)absorption. For inorganic cation transport, in contrast, solvent drag along claudin-2-formed paracellular channels appears feasible.

## Introduction

The concept of “solvent drag” in transepithelial transport was developed and advanced by Ussing and colleagues in the 1950s during their studies on water and solute transport across frog skin [2, 15]. In the following decades, the definition of solvent drag underwent many adaptations. Initially, the “drag effect” was not only envisioned as a “force exerted upon the solute molecules by the flow of solvent relative to the membrane” [24] but also, inversely, to be “created by the flow of sodium- and chloride ions through narrow pores” that provided a driving force for water transport [15]. Only 2 years later the concept was again inverted into “the force exerted upon the solute by the flow of solvent through the membrane [25].” In 1980, Whittembury et al. transferred the concept from transmembrane to paracellular transport when they studied the transepithelial transport of sucrose, inulin, and dextran and concluded “that solvent drags these solutes via the paracellular pathway [26].” Barry and Diamond [6] defined solvent drag as “a flux of solute coupled to water flow, caused by frictional interactions between solute and water traversing the membrane in the same channels and persisting even when solute concentrations immediately at opposite faces of the membrane are identical.”

The concept of solvent drag and its relevance to the transepithelial transport of different ion species and nutrients, especially in the renal proximal tubule and in the small intestine, was controversially discussed in the late 1970s and throughout the 1980s, but publication numbers declined during the 1990s. Thus, the majority of publications on solvent drag are from an era before the discovery of aquaporins and tight junction proteins. Although the total number of publications on solvent drag is small (in September 2022, a PubMed search for “solvent drag” and for (“solvent drag” AND (epithel* OR paracell*)) turned up 272 and 101 articles, respectively), the concept is firmly anchored in physiology textbooks.

In 1998, the first members of the claudin protein family were discovered and identified as the main constituents of tight junction strands [10]. Claudins were immediately recognized not only to be responsible for the formation of a paracellular barrier [14] but also for the charge and size selectivity of the paracellular pathway [1, 22]. The rapid developments in the understanding of a subset of claudins acting as paracellular channels within the tight junction strand over the past 25 years call for a reconsideration of the solvent drag concept.

## Coupling of water and solute transport

**Fig. 1:**
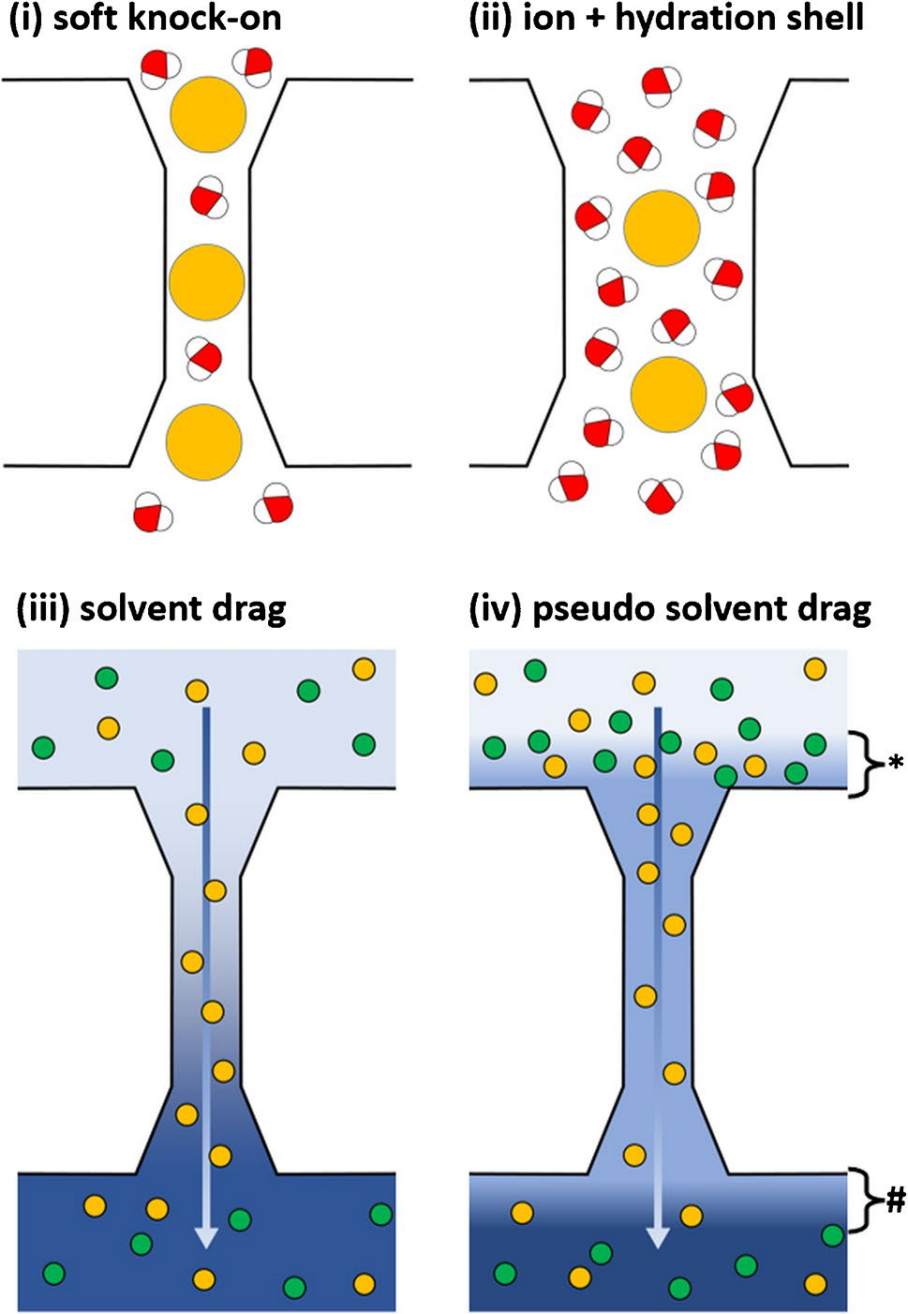
Potential mechanisms for the coupling of paracellular water and ion transport. **i** Single file soft knock-on mechanism: alternation of water and ion. **ii** Permeation of fully hydrated ions. **iii** Solvent drag: dragging of ions by the bulk movement of water. **iv** Pseudo solvent drag due to unstirred layer effects that cause local gradients. In **i** and **ii** ions (orange circles) and water molecules are depicted within a claudin-based paracellular channel. In **iii** and **iv** blue shading depicts overall osmolarity (sum of all osmolytes, gradient, e.g., generated by transcellular transport) outside and within a paracellular channel. Arrows indicate the direction of water movement, orange and green circles illustrate two different ion species, one being able (orange) and one not being able (green) to cross the barrier. *, #, unstirred layers with osmolyte enrichment on the *cis* face (*) and depletion on the *trans* face (#) of the barrier, respectively

Several mechanisms can be envisaged that would couple water and solute transport. Depending on the mechanism, the coupling ratio between water and solutes will differ (Fig. 1).

i. The lowest ratios include the single file alternating transport of ion and water, as, e.g., suggested for the permeation of potassium ions through a potassium channel [28] and dubbed “soft knock-on” mechanism [13], where the coupling ratio between water and ions would be 1:1.
ii. If solutes were simply holding onto their hydration shells while moving along their permeation pathway, the ratios would be between about five to up to some tens of H_2_O per solute molecule [5, 17]. However, mechanisms intermediate between (i) and (ii) are also conceivable, such as a partial loss of the hydration shell.
iii. For solvent drag, in the sense of dragging along the dissolved solute by the bulk movement of water, the coupling ratio should depend on the molar ratio of solute and solvent within that solution. One liter of extracellular fluid or primary urine at body temperature contains roughly 55 mol of water molecules, 145 mmol of Na^+^, 120 mmol of Cl^−^, and 5 mmol glucose, resulting in molar ratios of about 380 H_2_O/Na^+^, 460 H_2_O/Cl^−^, or 11,000 H_2_O/glucose molecule.
iv. Indirect coupling of water and solute transport may occur through a process called “pseudo solvent drag” [6, 7]. It is due to unstirred layer effects: movement of water along a bulk osmotic gradient will cause an enrichment of solutes close to the *cis* face of the epithelial cell layer and a dilution of solutes close to the *trans* face, thus establishing local gradients (* and #, respectively, in Fig. 1). These gradients provide the driving force for parallel solute transport. Conversely, the active transport of ions will result in local osmotic gradients and thus cause water movement. In both cases, no coupling ratio can be estimated, as it will depend on the thickness of the unstirred layers and thus on the stirring rate [6]. Pseudo solvent drag can either occur within one channel (as, e.g., demonstrated for water and Na^+^ transport by gramicidin; [18]) or through separate water and ion channels that act in parallel.

## The paracellular pathway: channel-forming claudins

The permeability of the paracellular pathway to electrolytes is provided by those members of the claudin family that form charge and size-selective paracellular channels. Current models of these channels do not provide any evidence for a 1:1 soft knock-on mechanism, i.e., mechanism (i) listed above.

The cation-selective channel-forming claudins 2, 10b, 15, and 21 differ in their Eisenman sequences, i.e., in their relative permeabilities P of the alkali metal ions Li^+^, Na^+^, K^+^, Rb^+^, and Cs^+^. These ions do not only differ in their ion diameter (Li^+^ being the smallest, Cs^+^ the largest) but also in the size of their hydration shells and the energy necessary to remove this hydration shell (Li^+^ largest, Cs^+^ smallest). Whereas Eisenman sequence I (P_Cs_ > P_Rb_ > P_K_ > P_Na_ > P_Li_) indicates that the ions hold on to their hydration shell while passing the selectivity filter of a channel, Eisenman sequence XI (P_Li_ > P_Na_ > P_K_ > P_Rb_ > P_Cs_) indicates that the hydration shell is removed [9]. Eisenman sequences observed for the permeation of alkali metal ions through claudin-based channels resulted in Eisenman sequence I for claudin-15, but Eisenman sequence IX to XI for claudin-2, -10b and -21 [11, 12, 23, 27]. This indicates a substantial removal of the hydration shell for the latter claudins, ruling out mechanism (ii) listed above, whereas for claudin-15, mechanism (ii) is a likely scenario.

For anion channel-forming claudins 10a and 17, the situation is less clear. However, as claudin-10a has been shown to be clearly selective for Cl^−^ over HCO_3_^−^ [8, 11], removal of the hydration shell at least for this claudin is likely. Dilution potential measurements on isolated proximal tubules from claudin-10a knockout animals indicate that claudin-10a provides the only relevant pathway for paracellular Cl^−^ transport in this nephron segment [8].

Paracellular water transport through specific claudins has been investigated in our lab by comparing total volume transport in claudin overexpressing and empty vector-transfected MDCK C7 cell layers exposed to electrolyte or osmotic gradients [3, 4, 19–21]. In all transfected cell clones, expression of other claudins and of aquaporins was quantified by Western blot. Clones with comparable expression levels were chosen for further experiments. In the presence of a mannitol-induced osmotic gradient, increased water transport was only observed in claudin-2 and -15 expressing cell layers, but not in claudin-10a, -10b, or -17 expressing cell layers [19–21].

In claudin-2 transfected cell layers, inducing an osmotic gradient not only induced H_2_O flux but, in parallel, increased unidirectional ^22^Na transport. The calculated coupling ratio amounted to ~ 1000 H_2_O per Na^+^ [21]. Conversely, an osmotically compensated Na^+^ gradient was able to induce water transport with a coupling ratio of about 500 H_2_O per Na^+^ ([21], Rosenthal, personal communication). Pore-lining amino acids I66 and S68 of claudin-2 were substituted with cysteines, and thiol-reactive reagents were added to sterically block the claudin-2 pore. Under these conditions, a decrease in both transepithelial conductance and water permeability was observed, again indicating, that Na^+^ and water were using the same pore [19].

For claudin-10b transfected cell layers, no water transport and hence no effects of osmotic gradients on Na^+^ flux or of osmotically compensated Na^+^ gradients on H_2_O flux were observed. The overall Na^+^ permeability of claudin-2 and -10b transfected cell layers, as calculated from dilution potential measurements, was similar. In the presence of an identical Na^+^ gradient, this should result in comparable unstirred layer effects, driving comparable water flux. As under these conditions, water flux was only observed in claudin-2 but not in claudin-10b transfected cell layers, it can be ruled out that the Na^+^ gradient driven water flux observed in claudin-2 transfected cell layers was predominantly pseudo solvent drag (mechanism iv above), i.e., transcellular water transport due to unstirred layer effects [21].

In claudin-15 transfected cell layers, Na^+^ flux was reduced in the presence of an osmotic gradient, and an osmotically compensated Na^+^ gradient did not elicit significant water movement. Thus, in the claudin-15-based pore, Na^+^ and bulk H_2_O movement appeared to interfere with each other. This assumption was supported by the finding that osmotically driven H_2_O flux increased in nominally Na^+^-free, NMDG-based solutions [20].

In summary, for the cation channel-forming claudins, only claudin-2 couples H_2_O and cation transport in a solvent drag-like manner. This is the transport depicted in Fig. 1iii. However, it differs from the classical definitions of solvent drag, as the pathway is charge and size selective and under physiological conditions restricted to H_2_O, Na^+^ and K^+^.

**Fig. 2:**
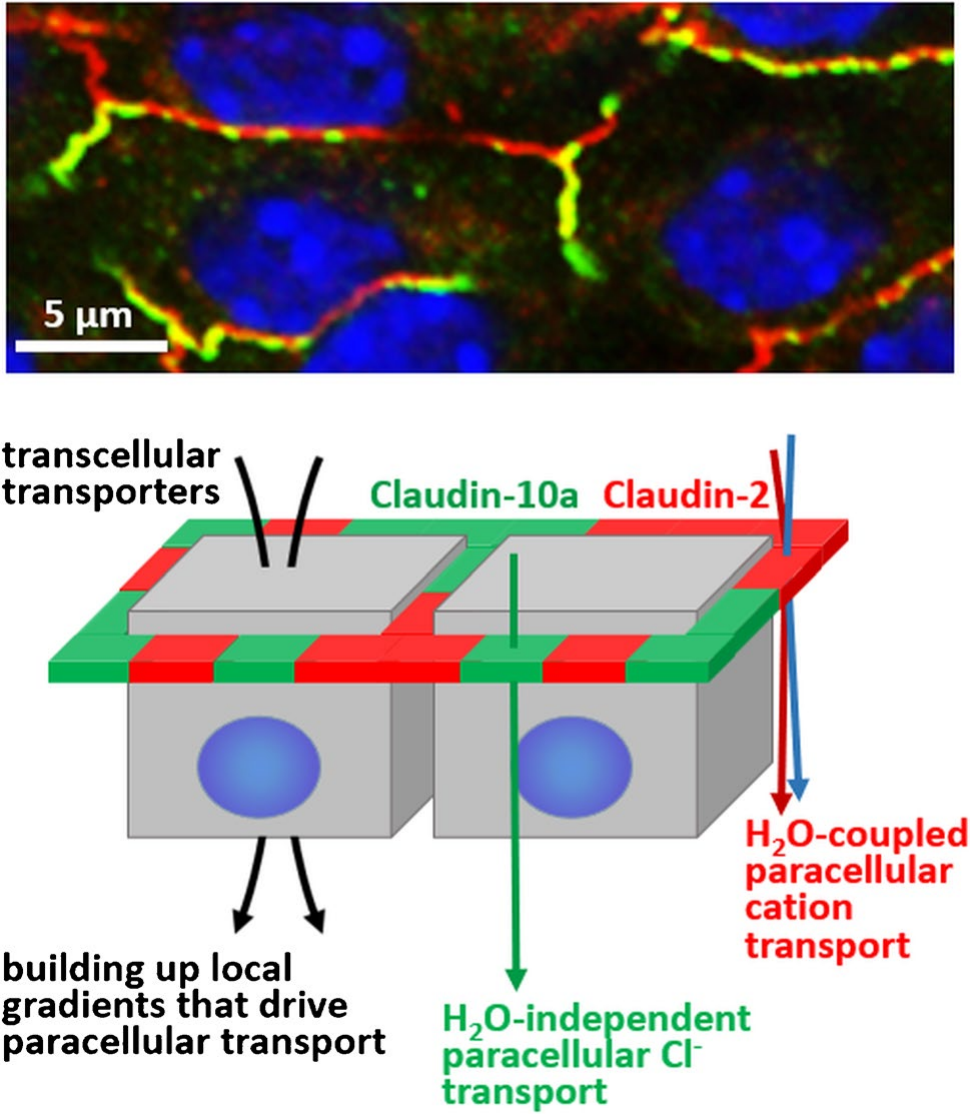
Ion and water transport in the proximal tubule. Upper: Immunofluorescence staining of claudin-2 (red) and claudin-10a (green) in a mouse proximal tubule showing the alternating arrangement of the two claudins in the tight junction (detail from [8]). Lower: Schematic drawing of trans- and paracellular transport in the proximal tubule. Transcellular solute transport leads to the build-up of local gradients through unstirred layer effects. These gradients drive the coupled H_2_O and cation reabsorption through the claudin-2-based paracellular channels by a solvent drag-like mechanism. The resulting local interstitial Cl^−^ depletion causes paracellular Cl^−^ reabsorption through the H_2_O impermeable claudin-10a-based paracellular Cl^−^ channels (pseudo solvent drag mechanism)

In contrast, paracellular anion transport, e.g., in the proximal tubule of the nephron, can only be indirectly coupled to H_2_O transport by a pseudo solvent drag mechanism (Fig. 2). Transcellular nutrient and bicarbonate reabsorption provides the osmotic gradient that drives H_2_O movement from the luminal to the interstitial face of the barrier and thereby reduces the basolateral Cl^−^ concentration. As claudin-10a provides a paracellular Cl^−^ pathway, Cl^−^ reabsorption occurs along this local concentration gradient towards the interstitium.

## The tricellular junction pathway

A claudin-independent pathway associated with paracellular H_2_O permeability is the pathway along the tricellular junction, i.e., the spots within a cell layer where three cells meet and that is tightened by a specific set of proteins. In MDCK C7 cells, knockdown of either of the major tricellular junction proteins, tricellulin or angulin-1 caused the opening of the tricellular junction and an indiscriminate permeability to water and to a wide variety of solutes such as Na^+^, Cl^−^, mannitol or even 4 kDa FITC-dextran [3, 4]. Thus, solvent drag is likely to occur along this route. In the intestine, this pathway has been found to play a role under inflammatory conditions, as a down-regulation of tricellular junction proteins and a translocation of macromolecules along the tricellular junction pathway has been observed in ulcerative colitis [16].

In the kidney, the relevance of this pathway is unclear as, to date, no pathophysiological conditions are known, under which tricellular junction proteins are down-regulated.

## Conclusion

In the proximal tubule of the nephron, alkali metal cations can pass the tight junctional barrier through claudin-2-based paracellular channels, whereas Cl^−^ moves along claudin-10a-based paracellular channels. Currently, among the channel-forming claudins, only claudin-2 is known to provide a pathway for a solvent drag-like transport which thus is selective for cations. In contrast, there is no molecular basis for anion (Cl^−^ or HCO_3_^−^) or organic solute reabsorption via solvent drag in the proximal tubule.

## Data Availability

Not applicable.
